# Design of Deployment Strategies to Monitor the Movement of Animals with Passive Electronic Devices

**DOI:** 10.3390/s21020326

**Published:** 2021-01-06

**Authors:** Laila D. Kazimierski, Jorge P. Rodríguez, Víctor M. Eguíluz

**Affiliations:** 1 Instituto de Física Interdisciplinar y Sistemas Complejos IFISC (CSIC-UIB), 07122 Palma de Mallorca, Spain; jorgeprodriguezg@gmail.com (J.P.R.); victor@ifisc.uib-csic.es (V.M.E.); 2 Instituto Mediterráneo de Estudias Avanzados IMEDEA (CSIC-UIB), 07190 Esporles, Spain

**Keywords:** animal movement, deployment strategies, radiotelemetry technique, central place movement model

## Abstract

Current animal monitoring systems have improved our knowledge of quantitative animal ecology. There are many electronic tracking technologies such as VHF/UHF telemetry, light-level geolocation, ARGOS satellite telemetry and GPS tracking. To reach the desired level of information retrieval requires the planning of adequate equipment effort and coverage, which depends on the properties of the system. We propose an equipment arrangement model consisting of a given number of receiver stations in a two-dimensional space in which the animals move according to a central place movement model. The objective is to characterize how the transmission of tracking data depends on the movement of the animals and the design of the equipment deployment: quantity and location of the receiver stations and their associated reception radius. We also implement the model using real trajectories of southern elephant seals and Australian sea lions publicly available online and tracked during the years 2010–2012. We characterize the data transmission based on different equipment configurations and we obtained analogous results to the theoretical model.

## 1. Introduction

The development of sensors for monitoring animals revolutionized the way of studying their behavior. Recent technological advances help us answer basic ecological questions such as when, how and where they move [1]. Among the most used electronic tracking methods are VHF/UHF telemetry, ARGOS satellite telemetry and GPS tracking [2,3,4,5,6,7,8,9], which allow us to obtain the location tracking of the animals. In addition to the position, we can measure environmental variables such as temperature or depth which can affect their movement. Furthermore, by combining location tracking with data acquired from inertial sensors such as accelerometers and gyroscopes, it is possible to identify when and where specific behaviors occur (feeding or escaping, among others). The transmitted information measured by the sensors placed on the animals is captured by receivers or is stored in memory for later transmission or downloaded once the equipment is recovered [10].

In particular, animals can be tracked remotely using electronic devices such as acoustic and satellite transmitters [11]. However, the monitoring methodologies and the used devices have several limitations that can bias our studies of animal movement. Those limitations include the amount of data that can be stored and can be sent restricted by the memory capacity or the battery power, and the fact that transmissions of data occur when the animals are within the reception radius of a receiver station. These aspects must be taken into account when designing a deployment of equipment to guarantee that the limited transmissions are optimized as much as possible [12].

To achieve efficient data transmission when working with animal monitoring, it is necessary to analyze the possible spatial configurations of the receiver stations. For example, this is particularly relevant for studies based on acoustic tracking. Acoustic tracking consists of monitoring animals tagged with acoustic transmitters using submerged omnidirectional receivers [13,14,15,16,17,18,19,20,21,22,23,24,25,26]. In addition to the number of receivers to be located and their spatial distribution, the temporal and spatial scales are relevant in the design of effective deployment strategies [27]. It must be considered that the equipment costs for acoustic tracking are very high. Therefore, it is useful to study different configurations to decide where to place the acoustic receivers for fish tracking [28]. As an example of this type of study, there is a work in which the “Receiver Efficiency Index” has been calculated with the aim of comparing the performance of two designed arrays of acoustic receivers to monitor fishes for optimal (>90%) detection of acoustic tags [29]. In addition, to interpret the results of the telemetry, the given biases of equipment deployment must be considered. For example, having more receivers at one site than at another or receivers with different reception radii can skew the results. This is why, in some cases, grid-like configurations are used [27,30].

The study of possible configurations for strategic locations of the receiver stations is also very valuable for VHF or UHF telemetry systems wherein the transmission of the collected data is transmitted when an animal is close enough to a receiver station [31]. Knowing the system and analyzing different distributions of the equipment will help us to design an efficient deployment and to get an idea about how to extend the network in case of having more receiver stations [32].

Acoustic receiver arrays arranged in two-dimensional grids can outperform linear arrays, based on the detection frequency of simulated animal movement [33]. There are some works in which telemetry is used to study animal movement where one-dimensional receiver lines or curtains and two-dimensional receiver grids have been used [34,35]. The problem of maximizing the covered area has also been studied extensively in recent years. For example, it has been proven that the fact of completely covering critical grids with a minimum number of receiver stations is NP-Complete [36,37]. Besides the configuration of the receiver stations, the number of tagged animals is also crucial [38].

We propose here an equipment deployment model consisting of a given number of receiver stations distributed in a two-dimensional space where the animals move following a central place movement model. We characterized how the transfer function of data depends on the movement of the animals and the receivers’ set-up. We study different scenarios regarding, on the one hand, the spatial arrangement of receiver stations (randomly locating them or near the animals’ homes or even making them mobile) and their associate reception radius and, on the other hand, regarding the movement of animals (simulating animal trajectories that tend or not to return to their homes).

This work is especially relevant to passive animal tracking, i.e., tagging animals with electronic devices that emit signals received by the receiver stations, because the acquired information depends on the spatial configuration of the receiver stations. Characterizing the transfer function allows us to make concrete decisions to study efficiently a given ecology system based on the animal movement, the equipment and its spatial distribution.

In addition to the simulated animal trajectories, we used movement data from two marine animal species to implement this model: southern elephant seals and Australian sea lions. Having some prior knowledge about the movement of the species under study can be useful in designing the deployment efficiently.

## 2. Model of Receiver Stations Deployment and Animal Movement

We model the distribution of equipment for animal monitoring in a two-dimensional space and the movement of animals to characterize data transmission rates. Animals move following a stochastic equation of motion and their tracking data are transmitted from attached data loggers to the receiver stations when they get in the reception radius of one of these stations. This model considers the following parameters: (i) the number of animals, the speed with which they move, and the attraction to their homes, (ii) the number of receiver stations, the reception radius and their location according to the deployment strategy. We aim to find the optimal equipment configuration for different movement parameters: number and location of the receiver stations and the radius of reception.

We have considered the following discrete-time stochastic equation describing the movement of each animal:

(1) $$\begin{array}{ccc} {\vec{x}}_{i}\left(t+1\right) & = & {\vec{x}}_{i}\left(t\right)+v\vec{\nu }\left(t\right)-a\left({\vec{x}}_{i}\left(t\right)-{\vec{x}}_{hi}\right) \end{array}$$

where ${\vec{x}}_{i}\left(t\right)$ is the position of animal *i* at time *t*, *v* is a parameter specifying the speed of movement,$\vec{\nu }\left(t\right)$ is a unit vector whose direction is drawn randomly from a uniform distribution, ${\vec{x}}_{hi}$ is a randomly selected position of the home for each animal *i* and a∈[0,1] is a constant parameter that measures the attraction to the home: a=0 leads to a random movement while, for a=1, the trajectories are points on a circle centered at the home with radius *v*.

**Figure 1:**
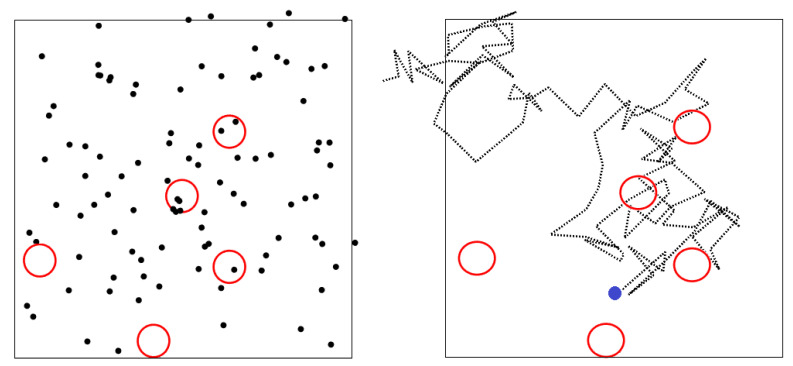
Illustration of the model. In the (**left**) panel, each black point represents an animal moving according to Equation (1) and the red circles represent the reception area of the receiver stations. The stations are located within the 1×1 enclosure. The homes of each animal are also within this space but are not included in this diagram. In each simulation, the value of the velocity and the value of the parameter attraction are fixed and are the same for all the animals. The (**right**) panel shows a trajectory for the case a=0 with the same arrangement of receiver stations shown in the left panel. The blue circle represents the home of that animal.

The home location ${\vec{x}}_{hi}$ and the receiver stations are located randomly in a two-dimensional space of dimensions 1×1, and we consider a population of *N* animals moving according to Equation (1) (Figure 1). At each time step, each sensor generates one package of data that stores the position of the animal carrying it. When an animal gets into the reception radius *r* of a receiver station, the sensors it has attached transmits the stored packages and deletes them from its internal memory. We measure the average number of transmitted packages per unit time per animal 〈T〉 and we study how it depends on the model parameters: the home attraction *a*, the reception radius *r* and the number of receiver stations. Finally, we analyze the performance of the model as a function of the spatial arrangement of the receiver stations (random or close to the homes) and whether the receivers are static or mobile.

## 3. Results

### 3.1. Results of Simulations

We divided this section into three parts, first characterizing the result of the simulations regarding how the transfer function changes depending on the model parameters, then characterizing the elapsed time between transmission events and, finally, considering the case of having mobile receiver stations.

#### 3.1.1. Characterization of the Transfer Function

**Figure 2:**
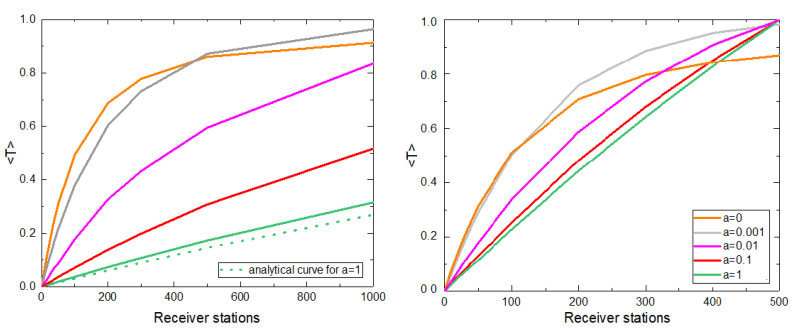
(**Left**) Average number of transmitted packages per unit time per animal 〈T〉 vs. the number of receiver stations for the random distribution case of the receiver stations. The dotted green curve indicates the theoretical probability of the transfer function for a=1. (**Right**) 〈T〉 vs. the number of receiver stations for the case where the receiver stations are distributed within the homes. In both graphs we show results averaged over 100 realizations, 20,000 time steps were considered, N=500, v=0.001, and r=0.01. Each curve corresponds to a different value of a=0, 0.0001, 0.01, 0.1, and 1 according to the color shown in the legend.

Considering the movement of animals according to Equation (1), we compute the average number of transmitted packages per unit time per animal 〈T〉 as a function of the number of receiver stations arranged randomly in space for different values of *a* (Figure 2, left).

We observe that 〈T〉 increases with the number of receiver stations, but this increase is influenced by the attraction to home *a*. First, the growth of 〈T〉 is reminiscent of a saturating exponential or exponential decay, with a first linear growth, more evident for large values of *a*, followed by a saturation, apparent for small values of *a*. Besides, given a fixed number of receiver stations, the 〈T〉 decreases with the attraction to home *a*. The extreme cases of *a* provide an interpretation for these curves. The case a=0 represents a random walk, such that sooner or later the animals encounter a receiver station and the data stored in their sensors is transmitted, which allows high values of 〈T〉, implying low data losses. In contrast, the case a=1 is almost static (when v≪r), such that if there is not a receiver station within a distance *v* from the home, data will never be transmitted, and it will be lost. For intermediate values of *a*, the animals move through smaller regions for higher *a* because they tend to return to their home site, with 1/a being the maximum allowed distance to the home along their trajectories.

For the case a=1, the movement can be approximated by a static movement (in the case of v≪r). Then, we estimate 〈T〉 as the probability that, at time t=0 (and hence at any time), a sensor attached to an animal falls within the interaction range of the receiver station. Thus, we estimate analytically 〈T〉 as the fraction of area covered by the reception zones of the receiver stations *f* (Figure 2 left, dotted green curve):

(2) $$\begin{array}{ccc} \langle f\rangle & = & 1-{\left(1-p\right)}^{x} \end{array}$$

where p=(π$r^{2})/A$, being *r* the reception radius of one receiver station, *A* the area of the square space and *x* the number of receiver stations.

**Figure 3:**
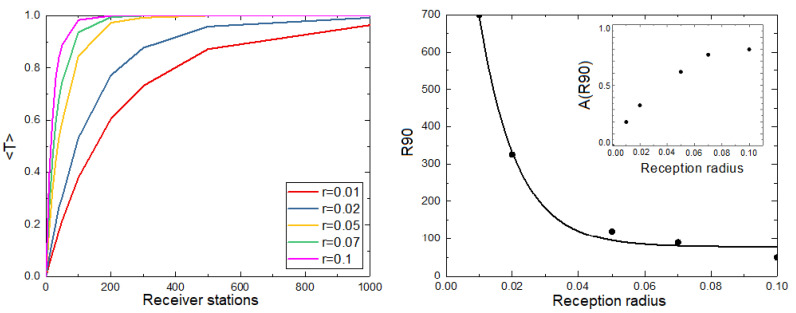
(**Left**) Average number of transmitted packages per unit of time and animal (〈T〉) vs. the number of receiver stations distributed in the space for a fixed value of *a* and different values of the reception radius *r*. Curves for different values of *r* are included. (**Right**) Number of receiver stations needed to receive an average of at least 90% of the total packages R90 as a function of *r*. The exponential fit of the form $y=yo+Ae^{\left(-(x-x0)/t1\right)}$ is included. The inset shows the fraction of the covered area for which 90% of the packages are transmitted as a function of the reception radius. For all graphs we show results averaged over 100 realizations. 20,000 time steps were considered, N=500, v=0.001, a=0.001.

One of the most relevant parameters of the model is the value of the reception radius *r*. Considering that the movement is characterized by a given value of *a*, we study the behavior of 〈T〉 with the number of receiver stations for different values of radius *r*, observing that the saturation of 〈T〉 with the number of receiver stations is faster for higher values of *r* (Figure 3 left). We quantify this saturation by introducing R90 as the number of receiver stations for which the system gets 90% of the total packages (Figure 3, right). As expected, R90 is lower as the value of *r* increases: the greater the reception radius, the smaller the number of reception stations required to cover most of the considered area. The value of R90 decays exponentially with the radius *r* (Figure 3 right). The inset of Figure 3 shows the fraction of the covered area (Equation (2)) when 90% of the packages are recovered as a function of the reception radius, showing that, with a smaller reception radius, it is necessary to cover less area to transmit the same number of packages. That is, it is more efficient to have many receiver stations with a small reception radius rather than a few stations with a large reception radius. In this last case, animals have successive interactions with them without transmitting large amounts of packages.

It is worth noting that for large values of *a*, the system does not saturate for the number of receiver stations considered and the growth of 〈T〉 approaches a linear regime (Figure 2, values a=0.1 and a=1).

**Figure 4:**
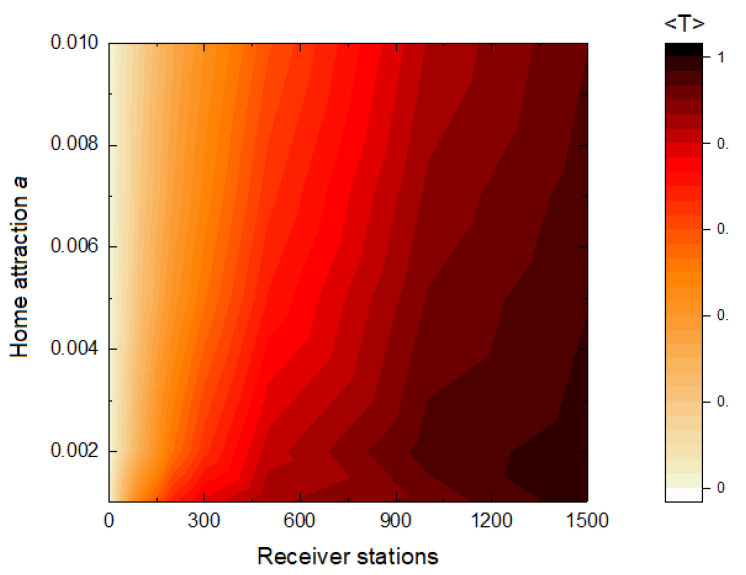
Average number of transmitted packages 〈T〉 per unit of time and animal as a function of the home attraction *a* and the number of receiver stations. Other parameter values: r=0.01, N=500 and v=0.001. 20,000 time steps were considered.

For small values of *a*, we can determine the number of receiver stations such that adding more receiver stations will not imply a significant growth of 〈T〉. In particular, this value could be R90. One way to observe this effect is through a phase diagram (Figure 4), where the color intensity represents the value of 〈T〉.

If we knew the site where the homes of the animals are located, we could place each receiver station within each home (no more than one station per home) instead of randomly distributing them and analyze, in this case, how the transfer function depends on the number of receivers and the attraction to the homes *a* (Figure 2, right). This distribution impacts directly in the case of animals with a large *a*, obviously increasing the probability of encounter, reaching maximum transfer when the number of receiver stations is greater than or equal to the number of tracked animals.

**Figure 5:**
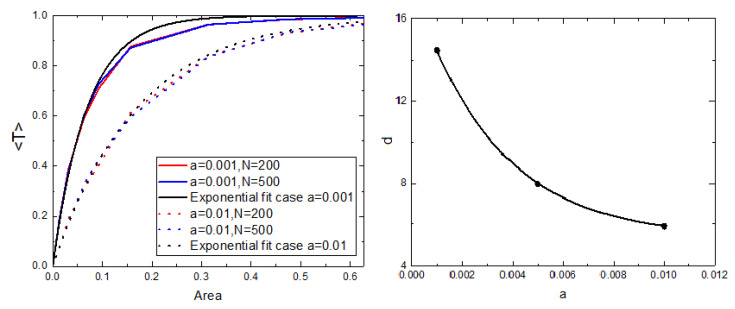
(**Left**) Average number of transmitted packages per unit of time as a function of the covered area for the case of random distribution of receiver stations. We include the cases a=0.001 and a=0.01 for N=200 and N=500. (**Right**) Exponent value *d* (obtained from exponential fits to the numerical simulations) as a function of *a*. In both graphs we show results averaged over 100 realizations, 20,000 time steps were considered, v=0.001 and r=0.01.

We analyze how the transfer function 〈T〉 depends on the number of receiver stations, their location and their reception radius in an integrated way by studying its dependence on the reception area covered by them (Figure 5, left). Here we include curves for two different values of *a* (a=0.001 and a=0.01) for two different number of sensors attached to animals (N=200 and N=500). The curves for each value of *a* (but different *N*) do not show significant differences, so we can generalize the behavior of each case as a function of the area. For a given value of the covered area, the system receives more packages if the movement of the animals is described by a lower value of attraction to the home *a*.

We fitted the two curves with an exponential function of the form $c(1-e^{-dx})$, from which we can extract values of the exponent *d* to characterize each growth. For the cases a=0.001 and a=0.01 we get the rates d=14.5±0.3 and d=5.91±0.07, respectively. The exponent *d* is lower as the attraction to home *a* is greater, and we find that this dependence is exponential (see Figure 5, right).

#### 3.1.2. Characterization of the Elapsed Time between Transmission Events

**Figure 6:**
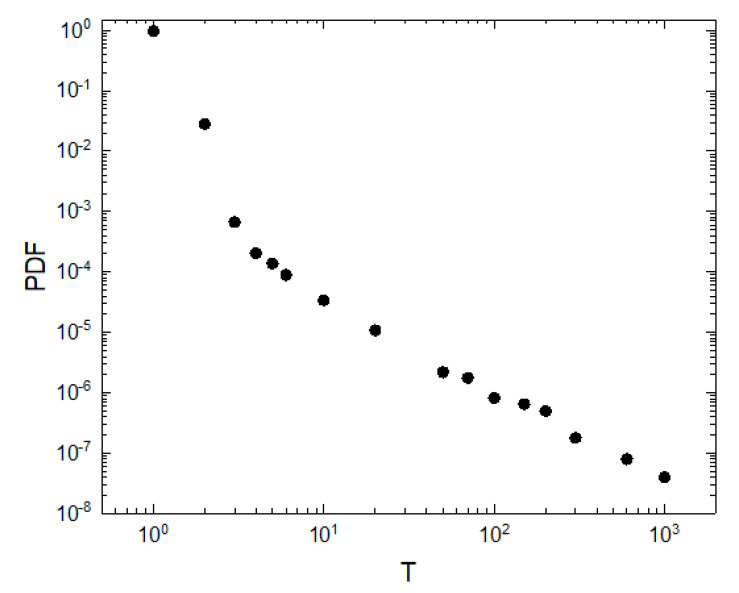
Probability density function of the inter-event times of consecutive interactions between a single animal and any receiver station for the case of 100 receiver stations. Both axes are shown in logarithmic scale. We show results averaged over 100 realizations, 100,000 time steps were considered, v=0.001, r=0.01 and a=0.001.

Another important aspect to analyze is the time elapsed between transmission events: how many steps do animals take before encountering a receiver station to transmit the stored packages. This is useful in determining which type of batteries (how long they last) and which type of memories (how much they can store) are necessary to guarantee the transmission of packages. We analyze the distribution of the inter-event times between the consecutive interactions between an animal and any receiver station and we show the probability density function of those inter-event times (Figure 6 for the case of 100 receiver stations, having found similar results with 20 and 50).

#### 3.1.3. Mobile Receiver Stations

**Figure 7:**
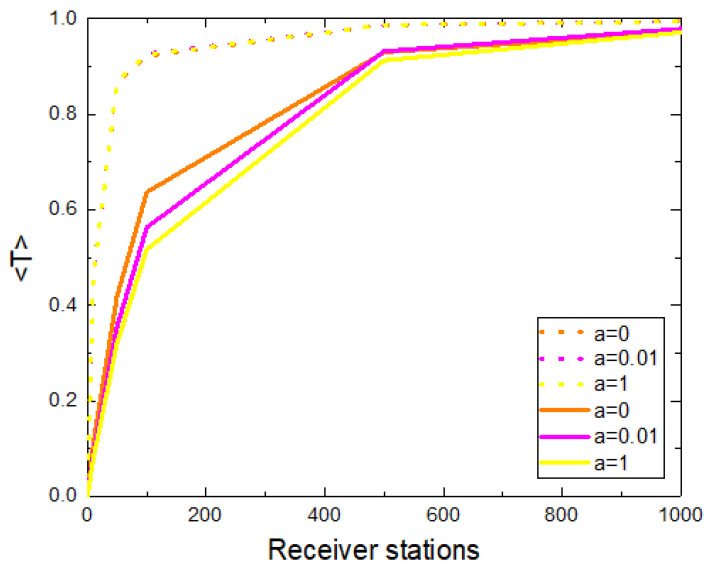
Average number of transmitted packages per unit of time and animal as a function of the number of receiver stations for the case of mobile receiver stations. The animals move with v=0.001. We include the scenarios where the receiver stations move (i) with the same velocity (solid line) and (ii) with higher velocity (dotted line, v=0.01). For each scenario, we include results for three different values of *a*. We show results averaged over 100 realizations, 20,000 time steps were considered and r=0.01.

We have analyzed the system considering mobile receiver stations instead of being fixed. We calculate the average number of transmitted packages per unit of time and animal as a function of the number of receiver stations that move randomly (see Figure 7). We consider the cases in which the velocity of the receiver stations is the same as that of animals and the case in which the receiver stations move with higher velocity. We find that the data transfer is more effective in the second case: for a given number of receiver stations, 〈T〉 reaches higher values in the case in which they move with a higher velocity than that of the animals. It is important to note that, in contrast to the case of fixed receiver stations (Figure 2 (left)), here a plateau is reached even for large values of *a*.

### 3.2. Model of Sensors in Real Trajectories

We implemented this model with real trajectories of southern elephant seals (SES) and Australian sea lions (ASL) publicly available online and tracked during the years 2010–2012 (data was sourced from Australia’s Integrated Marine Observing System (IMOS)—IMOS is enabled by the National Collaborative Research Infrastructure Strategy (NCRIS). In both cases, ARGOS was used to obtain the position of each animal).

**Figure 8:**
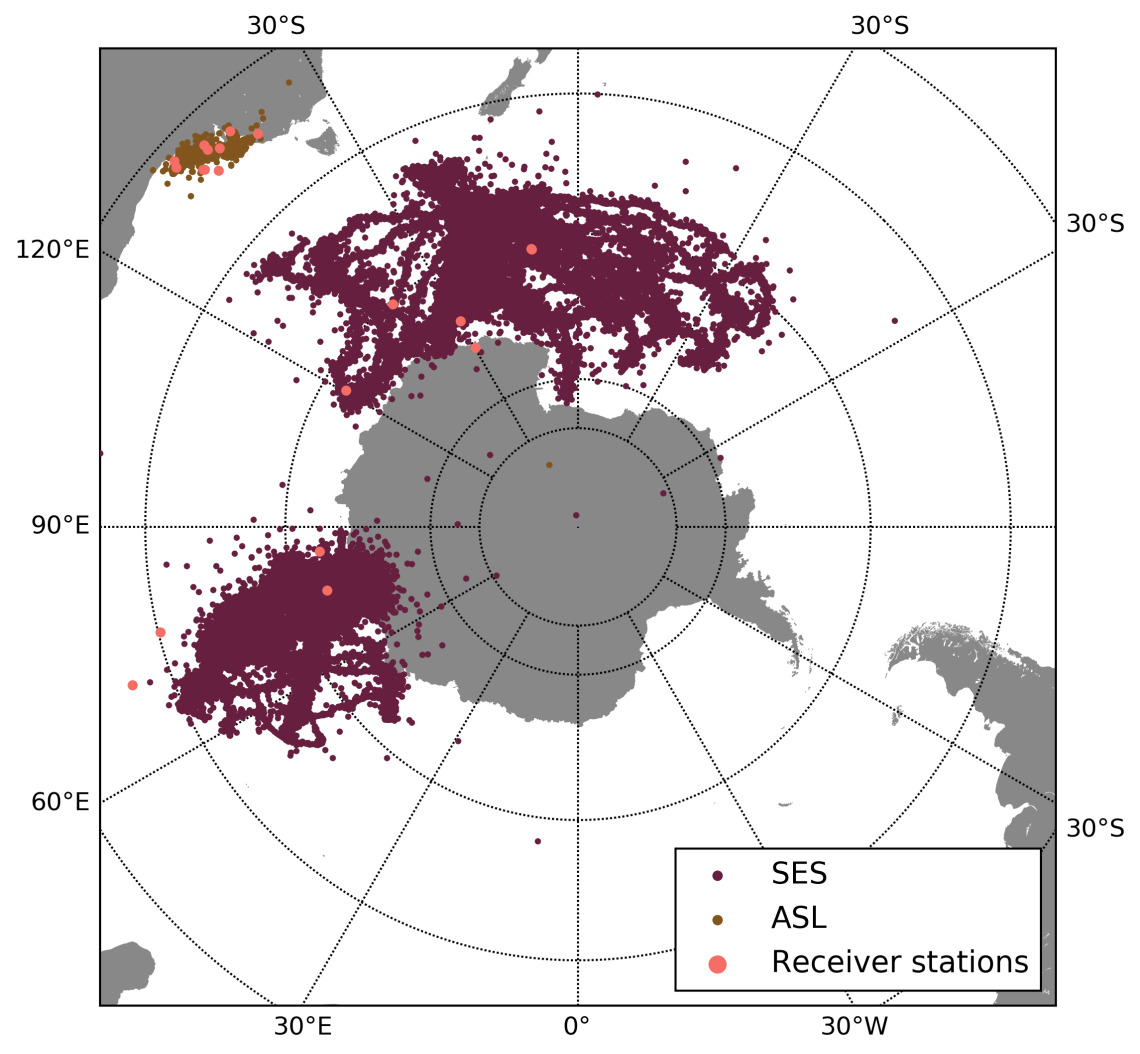
Trajectories of southern elephant seals (violet) and Australian sea lions (brown) tracked during the years 2010–2012. We also included 10 receiver stations randomly distributed for each species (pink dots).

In our model, each recorded position for each animal generates a new package. Ten receiver stations were located randomly for each species (see Figure 8, where we show the trajectories of each species and we included the ten receiver stations randomly distributed for each species (pink dots)). If an animal is near a receiver station, the stored packages are transmitted and deleted from the memory. That proximity was also associated with the reception radius parameter *r*. In this case, the Haversine distance was used to measure the distance between the animal’s position and the receiver station: if the Haversine distance between an animal and a receiver station in a given step is less than the radius, the stored packets are transmitted.

**Figure 9:**
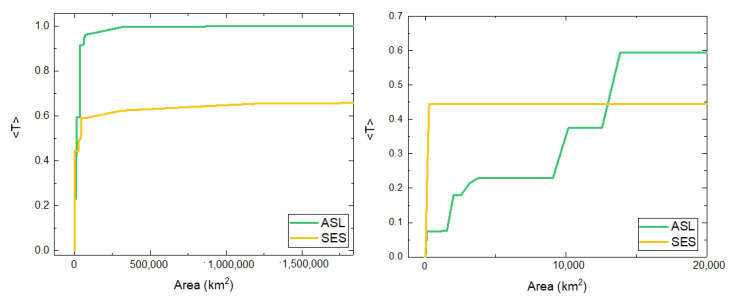
(**Left**) Average number of transmitted packages per unit of time as a function of the covered area for the case of random distribution of receiver stations for trajectories of SES and ASL. (**Right**) Same figure as the left panel, for total reception areas smaller than 20,000 km${}^{2}$. Since we associate the behavior of the SES with a smaller value of the parameter *a* than the one for the ASL, the colors of the curves are analog to the curves for small and great *a* shown in Figure 2.

Given that the SES travel greater distances than the ASL from their respective homes, we can compare the results of the implementation of the model to their trajectories by associating a higher attraction to the home parameter *a* for the case of ASL. We study, for each species, the transmitted packages 〈T〉 as a function of the covered area (see Figure 9 on the left panel and the detail on the right). For relatively low values of the area covered by receiver stations (right panel), the behavior of the transfer function for each species is analogous to those in the theoretical model taking into account that we associate the movement of the SES with a value of *a* lower than that of the ASL (Figure 2).

**Figure 10:**
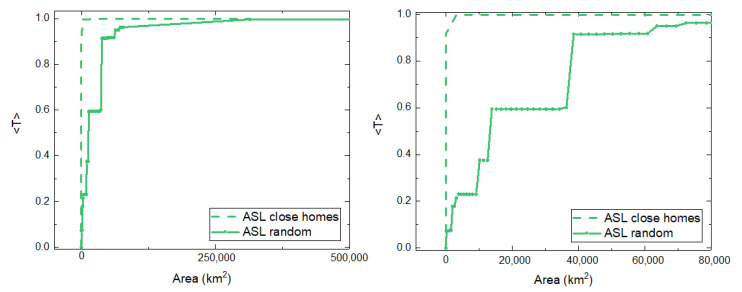
(**Left**) Average number of transmitted packages per unit of time as a function of the covered area for the case of ASL with a random distribution of receiver stations (line) and close to homes (dash). (**Right**) Same values as the left panel for total reception areas smaller or equal than 78,500 km${}^{2}$, which corresponds to the case of 10 receiver stations with a reception radius r=50 km${}^{2}$ each.

In the model, we show that for species with a higher value of *a* the impact of switching from a random distribution of receiver stations to a nonrandom one (receiver stations within the homes) is greater in the case of the species corresponding to the behavior modeled with a higher value of *a*. Analyzing the two distributions with real trajectories of ASL (associated with a large *a*), we find the same behavior (see Figure 10).

Analogously to the exponential fit done to the transfer function vs. the area for the random distribution of receiver stations for the model (see Figure 5, left), we fitted the real data. For the cases of SES and ASL, we obtained the value of exponent *d* of the exponential fits $d=\left(3.5\pm 0.95\right)\times 10^{-3}$ km${}^{-2}$ and $d=\left(4.3\pm 0.6\right)\times 10^{-5}$ km${}^{-2}$, respectively. Analogously to what the model suggests, the value of the exponent *d* decays with higher values of *a* (see Figure 5, right).

## 4. Discussion

We proposed a model to study how the data transfer function depends on the movement of animals and the spatial arrangement of the equipment considering the number of receiver stations, their location and their reception radius.

We analyzed this setup considering a central place foraging movement model [39,40,41], defined through a parameter that describes the attraction of the animals to their home, *a*, so we can describe species that perform a random movement in 2 dimensions (a=0) to those that stay close to their home (*a* close to 1). This makes our movement model suitable for simulating the behavior of land animals, in contrast to marine animals, where it would be a good approximation for movement along shallow waters (where it can be approximated in two dimensions). If diving behavior is considered, the movement model can be extrapolated to three dimensions since it only considers the distance, assuming that the space is isotropic.

The simplicity of the model allowed us to illustrate multiple scenarios, exploring how animals move, the places where it is more convenient to locate the receiver stations and the number of stations to be placed. Regarding the movement of the animals, future research should consider more realistic movement models, e.g., migratory movements, broad distributions of the displacements such as power-law or log-normal, Markovian dynamics, or collective movements, and study how movement patterns affect the deployment strategy. In fact, with our model we can study the case of animals that migrate between different sites, that is, consider the case where they have more than one associated home. If the animals have *m* associated homes, we can study the effect of this behavior by dividing the trajectories and associating them with *m* trajectories of different individuals.

This model allows us to study different scenarios by modifying the values of the most relevant parameters. There are several works in which arrays of over 50 receiver stations are used in acoustic tracking [26,42,43,44] and, also, close to 100 [45]. The phase diagrams like the one shown in Figure 4 allow us to study these scenarios for different radius values up to a maximum number of receivers given by the limitations of each study. Our framework can also be applied to design the spatial arrangement of animal monitoring with cameras [46,47]. Camera-networks are used to study and monitor animals in various types of habitats, and the optimal choice of the location to place the cameras is crucial [48]. However, the possible directional effect of the camera should be taken into account in the model.

Notice that data transmission may not be always successful, that is, the transmission may fail even if the animal is within the reception range of a station. This may be due, for example, to packages collisions or noise [28,49]. Our model does not consider a probability of transmission different than 1. However, it can be easily incorporated into simulations. For example, we can consider a probability of transmission equal to 0 if the animal is outside the range of a receiver station and greater than 0 (but less than 1) if the animal falls within that range. We can also consider that this probability increases as the animal approaches the station. We anticipate that these stochastic effects may match our results through the introduction of an effective reception radius.

## 5. Conclusions

We found that for a random arrangement of receiver stations in our model, 〈T〉 grows with the number of receiver stations, saturating into a plateau for small values of *a*; for the case of *a* close to 1 the growth is linear and the maximum values of 〈T〉 are relatively low for the number of receiver stations used.

If we place the receiver stations within the homes instead of randomly distributing them, 〈T〉 behaves linearly with the number of receiver stations but reaching, this time, values comparable to those reached by the animals whose movement is described with a small value of *a*. For certain system configurations (that is, number of receiver stations, their location in space and reception radius *r*), we can determine the number of stations needed to collect a given percentage of packages (in particular 90% for R90).

We study how the transfer function depends on the area covered by the stations. This allows us to characterize the growth of 〈T〉, for a given *r*, as a function of *a*, independently of the number of sensors placed on the *N* animals. We observe that this growth is exponential and we can determine the growth rate for each value of *a*. We find that, for a random spatial distribution of receiver stations, this rate decreases as *a* increases.

We also study how these results change if the receiver stations were mobile; in particular, if they move randomly in space. The transfer function saturates into a plateau in all cases (not so in the case of fixed receiver stations). It even reaches higher values of 〈T〉 for fewer stations in the case in which those move faster than the animals.

We implemented the model in real trajectories of southern elephant seals (SES) and Australian sea lions (ASL). We were able to study the dependence between the function of transmitted packages and the covered area by the receiver stations. In particular, we found similar results to those observed in the model. On the one hand, considering relatively low values of the area, the random arrangement of the receiver stations is more efficient for the case of species described with a small home attraction. On the other hand, we find that a nonrandom arrangement of the receiver stations favors the transfer function in the case of a species modeled with a larger home attraction. Finally, the exponential fit made to the transfer functions allows us to characterize the growth as a function of the area for the different species, obtaining a value of the exponent that corresponds to what is expected in each case.

## Data Availability

Code and data are public and available at https://gitlab.com/lailakaz1986/model_sensors.
